# Identification, Characterization, and Palynology of High-Valued Medicinal Plants

**DOI:** 10.1155/2013/283484

**Published:** 2013-06-10

**Authors:** Hina Fazal, Nisar Ahmad, Bilal Haider Abbasi

**Affiliations:** ^1^Pakistan Council of Scientific and Industrial Research (PCSIR) Laboratories Complex, Peshawar 25120, Pakistan; ^2^Department of Biotechnology, Faculty of Biological Sciences, Quaid-i-Azam University, Islamabad 45320, Pakistan

## Abstract

High-valued medicinal plants *Achillea millefolium, Acorus calamus, Arnebia nobilis, Fumaria indica, Gymnema sylvestre, Origanum vulgare, Paeonia emodi, Peganum harmala, Psoralea corylifolia, Rauwolfia serpentina*, and *Vetiveria zizanioides* were identified with the help of taxonomical markers and investigated for characterization and palynological studies. These parameters are used to analyze their quality, safety, and standardization for their safe use. Botanical description and crude drug description is intended for their quality assurance at the time of collection, commerce stages, manufacturing, and production. For this purpose the detailed morphology was studied and compared with the Flora of Pakistan and other available literatures. Here we reported the pollen grain morphology of *Origanum vulgare, Paeonia emodi, Psoralea corylifolia*, and *Rauwolfia serpentina* for the first time. Similarly the crude drug study of *Gymnema sylvestre* (leaf), *Origanum vulgare* (aerial parts), *Paeonia emodi* (tubers), and *Peganum harmala* (seeds) was also carried out for the first time.

## 1. Introduction

The use of herbal medicine for the treatment of diseases and infections is a safe and traditional therapy [1]. In developing countries, medicinal plants are attaining greater importance in the primary health care of individuals and communities. Medicinal plants form a large group of economically important plants that provide the basic raw materials for indigenous pharmaceuticals [2, 3]. The drugs are quite often adulterated or substituted by other low-quality plant material before harvesting and during handling and storage. Therefore, for attaining the quality assurance of herbal formulation in any system of medicine, emphasis should be given for good harvesting practices (GHP), good laboratory practices (GLP), and good manufacturing practices (GMP) [4]. One of the objectives of the authentication of the crude drug is to check its adulteration, which is useful in promoting the usage of genuine drugs contributing to the health care of human society. Herbal remedies used as medicine is often available in fragmentary form, and identification relies on microscopical rather than macroscopical evaluation. But it is quicker to find out the identity of a crude drug from its anatomy than from its chemistry. For proper identification and standardization of crude drugs, accurate anatomical, and morphological description is necessary, and this description must take into account all the diagnostic features. The authenticity of the crude drug is established by reference reported in the monograph and official pharmacopoeia. The evaluation of crude drug involves a number of methods such as organoleptic, microscopic, chemical, physiological and biological. Organoleptic evaluation includes shape, size, odor, taste, texture, and color of crude drug along with external marking. On the basis of gross morphology, drugs may be grouped as leaves, bark, root, rhizome, and so forth. Microscopic features such as spines, trichomes, spores, and epidermal structures may be examined which are used as diagnostic features in the identification of plant drugs. Taste and odor are extremely valuable tests when carried out carefully. Similarly for microexaminationl characteristics such as starch, calcium oxalate, epidermal cells, trichomes, fibers, and vessel stone cells are examined. 

The selection of plant material would be based on availability of certain features which provide taxonomic evidence for their evaluation. The plant characters observed most commonly relate to gross morphology or at least to features readily by external examination with the naked eye. Morphological characters play significant roles for correct identification, characterization, and delimitation of taxa on the basis of available markers. The use of plants as therapeutic agents is paramount in virtually all systems of traditional medicine and especially in Unani medicine. It is necessary to develop a mechanism for quality assurance of plants used as drugs in these medical systems [5]. Quality assurance of herbal medicine seems to be little explored, and that is why standardization and authentication have taken a serious turn. It, therefore, seems obligatory to procure these indigenous drugs and certify their identity by taxonomic and chemical methods. 

Pollen morphology is conducted as an aid to the morphological study and a significant tool for modern taxonomist for the delimitation of species. Pollen characters are useful in solving complicated problems of interrelationships between various taxa and assessment of their status in the classification, particularly with reference to the families, subfamilies, tribes, genera, species, and subspecies. Mature pollen grain size, exine sculpturing, and number of pores are the most distinctive features [6, 7]. Palynological data has been useful at generic and specific level [8]. This analysis also helps in qualitative analysis of drug powder and the correct identification of drug. It plays an important role in our daily life as well. Aerobiology has received much attention due to its wide application in allergology, forestry, agriculture, horticulture, archaeology, and plant geography [9, 10]. 

From the reported literature, it is evident that *Achillea millifolium*, *Acorus calamus*, *Arnebia nobilis*, *Fumaria indica*, *Gymnema sylvestre*, *Origanum vulgare*, *Paeonia emodi*, *Peganum harmala*, *Psoralea corylifolia*, *Rauwolfia serpentine,* and *Vetiveria zizanioides* are widely used in herbal medicines. They contain remarkable active constituents responsible for their biological activities, but these plants are not thoroughly investigated so far. Therefore, the main objective of the current study was to standardize these plants in line with the standards of quality detailed in the British Herbal Pharmacopoeia, World Health Organization (WHO) monographs, and other official publications.

## 2. Material and Methods

Fresh plants were collected from different localities of Khyber Pakhtunkhwa, Pakistan, for taxonomic studies both from wild and cultivated sources. Efforts were made to collect the plants when they were in flowering and fruiting periods for their correct botanical identification. Those plants which were not locally available, different herbaria of Pakistan were consulted for them like Herbarium PCSIR Laboratories Complex, Peshawar (PES), Quaid-i-Azam University herbarium, Pakistan, Museum of Natural History, Islamabad and Peshawar University herbarium.

The plants were identified by consulting Fascicles of Flora of Pakistan, Flora of British India [11], and other available literature. The detailed morphological characteristics of the species were established from fresh samples, herbarium specimen, or by consulting the literatures. In order to ensure a methodical study of the material obtained, herbarium samples were prepared according to the method of Fazal et al. [12] and stored at the Herbarium, PCSIR Laboratories Complex, Peshawar (PES), for future reference.

### 2.1. Microscopic Studies Crude Drugs

Macro- and microscopic characters of the crude drugs of *Achillea millefolium*, *Acorus calamus*, *Arnebia nobilis*, *Fumaria indica*, *Gymnema sylvestre*, *Origanum vulgare*, *Paeonia emodi*, *Peganum harmala*, *Psoralea corylifolia*, *Rauwolfia serpentine,* and *Vetiveria zizanioides* were studied as per Wallis [13] and Trease and Evans [14]. For microscopic characters, the plant material was finely powdered. Several samples were prepared in different mounting media, especially in chloral hydrate solution and water. For this purpose, one or several drops of the medium were placed in the center of a clean slide. A small amount of test material was sprinkled on this fluid. The precleaned cover slip, held with a tweezers, was placed on slide starting from one edge. This edge should first make contact with the mounting medium; the glass is then lowered into place. This permits air bubble to escape. The slide was then observed under compound microscope, and the characters were recorded and compared with different pharmacopoeias and monographs for confirmation.

### 2.2. Organoleptic Evaluation/Macroscopic Studies

The organoleptic features of the plant were examined by using sensory organs or by using a magnifying glass. For organoleptic properties a panel of 9 members with different ages ranging from 23 to 30 years with 3 females and 6 males who were familiar with such characteristics was selected for analysis. The organoleptic properties of these medicinal plants were including color, odor, taste, external margins, apices, texture, external and internal marking, fracture, shape and size. Three groups of members were allowed to scale (1–9) these sensory properties. Descriptions for each score were as follows: 1 = good taste, 2 = bitter taste, 3 = moderate taste, 4 = color, 5 = good aroma, 6 = bad aroma, 7 = moderate aroma, 8 = smooth surface and 9 = rough surface. The Organoleptic test was performed in the Medicinal Botanic Centre (MBC) at the Pakistan Council for Scientific and Industrial Research (PCSIR) Labs Complex, Peshawar, Pakistan under suitable light conditions at temperature of 25 ± 2. The result from three independent groups was analyzed by Statistix 8.1 software for mean values and probability.

### 2.3. Plant Material for Palynological Study

**Figure 1:**
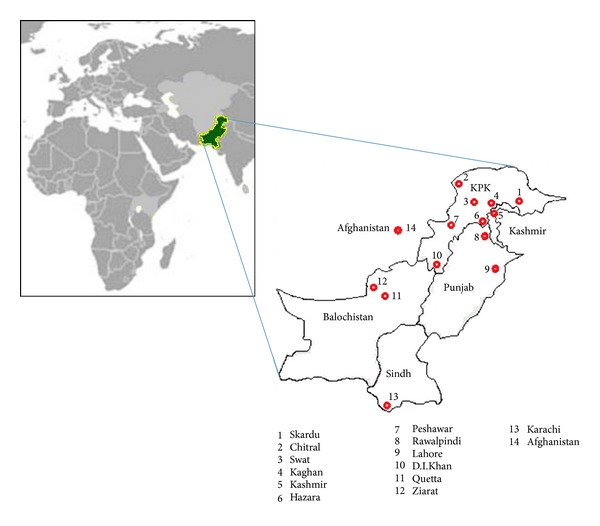
Map showing the areas of distribution of medicinal plants in Pakistan used in the present studies.

Flowers and pollens of* Achillea millefolium* and *Paeonia emodi* were collected from Kaghan, *Fumaria indica *from Haripur, *Peganum harmala *from Lakki Marwat, *Origanum vulgare, Psoralea corylifolia, *and *Rauwolfia serpentina *from experimental farm PCSIR Laboratories Complex, Peshawar (Figure 1). The flowers were dried, and few of the plant pollens were freshly isolated and stored in vials containing acetic anhydride.

#### 2.3.1. Methodology

Pollens samples were isolated from their anthers with the help of forceps and needle under a dissecting microscope. The pollens were prepared for scanning electron microscopy by standard method described by Erdtman [15]. The pollen grains, suspended in acetic anhydride, were placed on slide. They were crushed with the help of a glass rod, and the debris was removed with the help of a needle. The drop of acetic anhydride containing the suspended pollen grains was poured on the metal stub. The stub was placed for drying for approximately 30 minutes and then was coated with gold in a sputter chamber (ion-sputter JFC-1100) with coating restricted to 150°A [16]. Scanning electron microscopy (SEM) examination was performed on Jet microscope JSM-T200. The measurements were based on 10–15 readings for each specimen. Pollen characters like shape, class size aperture, and exine ornamentations were studied. Microphotographs were taken at the Centralized Resource Laboratories, University of Peshawar, Pakistan. 

### 2.4. Statistical Analysis

The data for statistical analysis was collected from three independent experiments. Mean values were obtained by using Statistix software (8.1). Mean values are significantly different when *P* < 0.05.

## 3. Results

**Figure 2:**
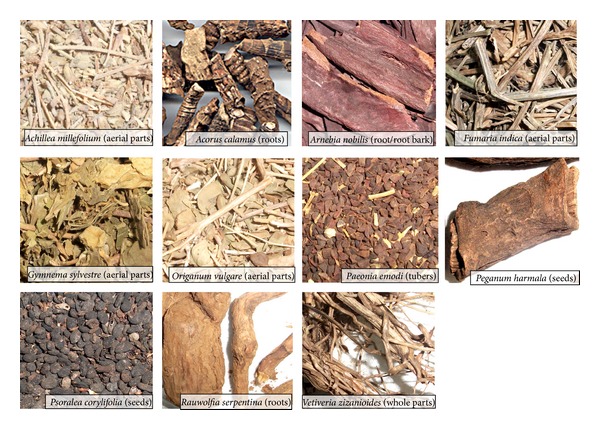
Different parts of high valued medicinal plants used in different herbal formulations. Aerial parts of *Achillea millefolium*, roots of *Acorus calmus*, roots of *Arnebia nobilis*, aerial parts of *Fumaria indica*, aerial parts of *Gymnema sylvestre*, aerial parts of *Poeonia emodi*, seeds of *Peganum harmala*, seeds of *Psoralea corylifolia*, roots of *Rauvolfia serpentine* and whole parts of *Vetiveria zizanoides*.

In the present investigation, the detailed taxonomic, pharmacognostic, and biological studies of eleven medicinal plants were carried out. These plants are *Achillea millefolium, Acorus calamus, Arnebia nobilis, Fumaria indica, Gymnema sylvestre, Origanum vulgare, Paeonia emodi, Peganum harmala, Psoralea corylifolia, Rauwolfia serpentine, *and *Vetiveria zizanioides *(Figure 2).

### 3.1. Morphological, Palynological and Crude Drug Description

The detailed taxonomic and morphological studies of eleven medicinal plants along with their local names, family, distribution, part used, organoleptic characters, macro- and microscopical description of plant material of interest, and pollen grain morphology were carried out. The results have been arranged in alphabetical order by species name as shown in Table 1.

## 4. Discussion

The detailed taxonomical description is intended for quality assurance at different stages of processing of crude drug for product manufacturing. For this purpose, the morphological studies were carried out through identification of taxonomic markers. For confirmation, these characters were compared and confirmed by consulting Fascicles of Flora of Pakistan Nasir [17–19]; Nazimuddin and Qaiser [20]; Qaiser [21]; Ali [22, 23]; Ghafoor [24]; Hedge [25]; Jafri [26]; Li et al. [27]; and other available literatures. 

In present study, pollen grain morphology of *Origanum vulgare, Paeonia emodi, Psoralea corylifolia,* and* Rauwolfia serpentina* is reported for the first time. The scanning revealed that pollen grains of these plants are scarbate, foveolate, and tectate with smooth surfaces. The pollen grains of *A. millefolium* were found to be compositous, that is, echinate and tectate with spiny exine and 3 pores. The pollens of *F. indica* were in line with the results of Perveen and Qaiser [8] namely, they are triporate, oblate-spheroidal, pore more or less circular, operculate, annulate sexine thicker than nexine, fossulate-foveolate, and exine is 2.5 *μ*m thick. The pollens of *P. harmala* are suboblate-subprolate and tricolporate, ornamentation is striate-rugulate and exine 1.2 *μ*m, sculpturing reticulate, consisting of lumina and muri, perforate, pit or hole diameter less than 1 *μ*m [28].

The pharmacognostic studies include the collection/procurement of various parts of these medicinal plants, their identification, standardization, and authentication through various taxonomic markers and macro- and microscopic characters. The evaluation of crude drug which eventually enters the commercial market is obviously of considerable importance. Companies involved in the crude drug sale generally avoid special recommendations on the use of a product. There are no applicable standards of quality for crude drugs, and they are not usually standardized with respect to the concentration of active constituents. For these reasons it is necessary to subject such crude drugs to various standards of quality, purity, and safety, if acceptable consumer usage is to be achieved.

In the present study, crude drug study of leaf of *G. sylvestre, *aerial parts of* O. vulgare,* tubers of *P. emodi,* and seeds of* P. harmala* was reported for the first time. Previously Hyde [29] performed the crude drug study of aerial parts of *A. millefolium* indicating that fragments of leaves, small flower heads, and stem pieces are visible, while microscopic characters include trichomes long, uniseriate, with pointed terminal cell, glandular trichomes compositous, with about 4 pairs of cells. Leaf epidermal cells are elongated with sinuous anticlinal walls, while stomata are anomocytic. The unpeeled drug of *A. calamus* is covered with a thin brown cork. The peeled drug is creamy yellow in color with fewer root scars. The section has a large stele separated by a yellowish line from the cortex. Khatoon et al. [30] and Arora et al. [31] studied the root and root bark of *A. nobilis*, which indicated that they are furrowed and they are too deep that cylindrical form of root is lost and irregular segments are formed.

The authentication of the crude drug material is necessary for many reasons. Firstly, a new source of drug material must be specified if it is to be used commercially. Secondly, people using plants for medicine must know the source of their material; otherwise they may not be able to gain their objectives, with failure to duplicate pharmacological results obtained with extracts from subsequent lots of material. This is a real problem in cases where the source of the material is not authenticated. Thirdly, when a drug has become an article of commerce, it becomes adulterated with unsuitable ingredients; here, microscopy can be used to give an indication of purity. 

## Figures and Tables

**Figure 3:**
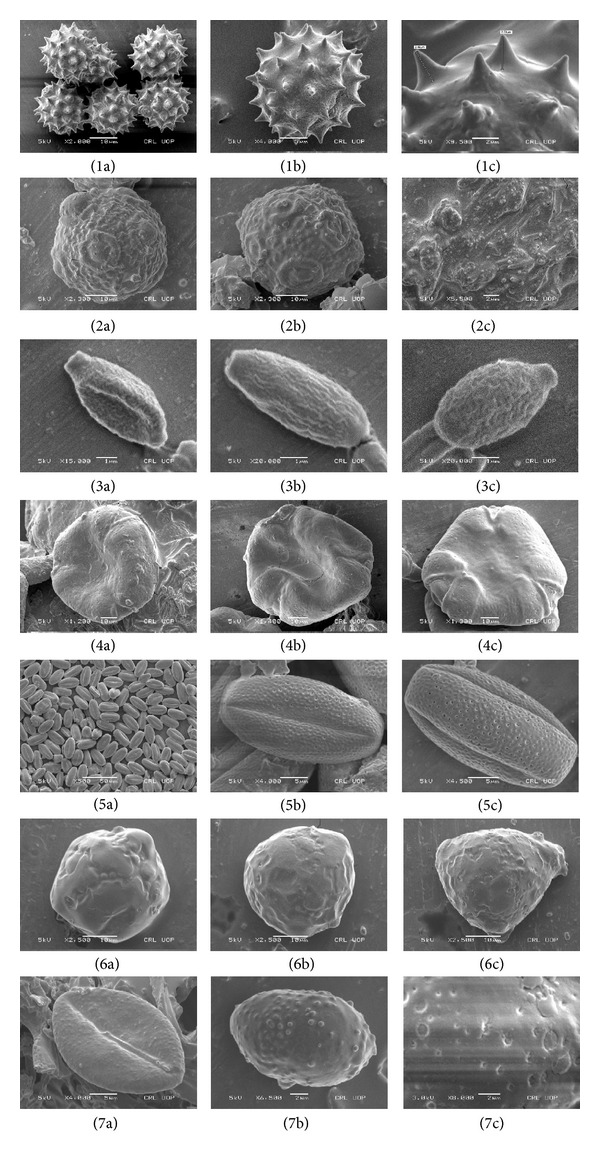
Palynological studies of pollen grains of seven high valued medicinal plants. (1a)–(1c) Pollen morphology of *Achillea millefolium* (2a)–(2c) *Fumaria indica* (3a)–(3c) *Origanum vulgare* (4a)–(4c) *Paeonia emodi* (5a)–(5c) *Peganum harmala* (6a)–(6c) *Psoralea corylifolia* (7a)–(7c) *Rauwolfia serpentine*.

**Table 1:** 


(1) Botanical name	*Achillea millefolium* L.
Local name	Baranjasaf
Family	Asteraceae
Distribution in Pakistan	Swat, Hazara, Kaghan, and Peshawar
Plant material of interest	Aerial parts
Organoleptic	Fragrant odour, taste is bitter
Macroscopic characters	Green fragments of the pinnate leaves; the downy segments are linear and finely pointed. Many small flower heads are surrounded by long felted hairs. Stem pieces are ribbed, rounded, pithy, downy, and green to violet red.
Microscopical characters	Trichomes are long, uniseriate, with pointed terminal cell. Glandular trichomes are compositous, with about 4 pairs of cells. Leaf epidermal cells are elongated with sinuous anticlinal walls. Stomata anomocytic. Calcium oxalate is absent.
Pollen grain morphology	Pollen grains are compositous, that is, echinate and tectate about 30 *µ*m in diameter, with a coarsely spiny exine and 3 pores; spines are more than 1 *µ*m (Figure 3).
Traditional uses	Used to relieve fever, delivery pain, and diarrhea and is hepatoprotective.

(2) Botanical name	*Acorus calamus* L.
Local name	Bach
Family	Araceae
Distribution in Pakistan	Chitral, Peshawar, Kashmir, Rawalpindi, and Poonch
Plant material of interest	Rhizome.
Organoleptic	Pungent, taste is bitter
Macroscopic characters	Rhizome is covered by brown cork and deeply wrinkled longitudinally. It bears triangular leaf scars and hair-like fibers on the upper surface. The lower surface has small root scars. The peeled drug is cream yellow in color. Fracture is sharp producing a granular, white, and spongy surface.
Microscopical characters	Powder is yellowish white, consisting of oval-shaped parenchymatous cells. Yellow-brown oleoresin, and starch grains are present. A few xylems are present.
Traditional uses	Used to cure diabetes and high blood pressure.

(3) Botanical name	*Arnebia nobilis* Reichb. f
Local name	Ratanjot
Family	Boraginaceae
Distribution	Endemic species of Afghanistan
Plant material of interest	Root/root bark
Organoleptic	Smell aromatic, tasteless
Macroscopic characters	Root color is purple brown, twisted, deeply furrowed, and irregular. Length of segments is 5–10 cm and 3–6 cm in diameter. It is covered with papery layers of the same color.
Microscopical characters	Outermost xylem with broad vessels and innermost with groups of radially arranged narrow vessels while the middle region is occupied by alternate rings of clusters of broad and narrow vessels and presence of pith.
Traditional uses	Used to cure jaundice, kidney pain, wound healing, and diarrhea.

(4) Botanical name	*Fumaria indica* (Hausskn.)
Local name	Shahtra
Family	Fumariaceae
Distribution in Pakistan	Swat, Hazara, Peshawar, Rawalpindi, Lahore, Ziarat, Karachi, and Hyderabad
Plant material of interest	Aerial parts
Organoleptic	Taste is slightly bitter
Macroscopic characters	Fragments of green glabrous leaves. Flower petals are shrunken and red-violet. Fruits are flattened about 2 mm in diameter and green containing one seed. Stem pieces are light green or brown in color, ribbed, and hollow.
Microscopical characters	Leaf epidermal cells with anomocytic stomata. Calcium oxalate is absent. Pollen grains spherical with a pitted exine.
Pollen grain morphology	Triporate, Oblate spheroidal, pore more or less circular, operculate, fossulate-foveolate, and exine 2.5 *μ*m thick (Figure 3).
Traditional uses	Juice of the plant is given in fever, removing worms from abdomen, diabetes, bladder infection, piles, and allergy. Also used in the treatment of goiter, as diuretic and antihelmintic.

(5) Botanical name	*Gymnema sylvestre* Retzius Spreng.
Local name	Gurmar
Family	Asclepiadaceae
Plant material of interest	Leaf
Organoleptic	Odourless, taste is slightly bitter
Macroscopic characters	Stems and leaves hairy stem are hollow, ridged, golden brown from the outer side thin bark and green and from the inside. Petiole 3–12 mm; leaf obovate to ovate, 3–5.5 cm, thick papery, adaxially pubescent to glabrous, abaxially, and glabrous.
Traditional uses	Leaf juice is used in eye diseases and snakebites. It is also used to remove the effect of wine and other narcotic drugs.

(6) Botanical name	*Origanum vulgare* L.
Local name	Satar
Family	Lamiaceae
Distribution in Pakistan	Chitral, Swat, Hazara, Malakand, Kashmir, and Rawalpindi
Plant material of interest	Aerial parts
Organoleptic	Aromatic, taste is like *trachyspermum. *
Macroscopic characters	Stems are branched, thinly to densely pilose with spreading hairs, glabrous, purplish, or green. Leaves are simple, entire, ovate, 5–30 mm, gland-dotted, apex acute, or obtuse, with scattered hairs, petiolate.
Microscopical characters	Trichomes, green oval cells, epidermal and parenchymatous cell, and pollen grains are present.
Pollen grain morphology	Profusely reticulate, lumina, and muri are present.
Traditional uses	Used as fresh fodder and for washing utensils, as diuretic, and is also used in toothache and earache.

(7) Botanical name	*Paeonia emodi* Wall. ex Royle
Local name	Mamaikh
Family	Paeoniaceae
Distribution in Pakistan	Chitral, Swat, Hazara, and Ziarat
Plant material of interest	Tubers
Organoleptic	Aromatic, taste is bitter.
Macroscopic characters	Tubers are cylindrical, straight, or curved, 5–9 cm in length, 1.2–2.2 mm in diameter, externally dark brown with thick bark, internally light brown, bark 1-2 mm in diameter; ridges and furrows are present; few furrows are too deep.
Microscopical characters	Powder is yellowish to pink in color, parenchymatous cells are visible, starch granules are abundant, and calcium oxalate crystals, oil globules, and reticulate vessels are present.
Pollen grain morphology	Sculpturing scarbate with smooth surface.
Traditional uses	Its powder is used in dysentery and chronic diarrhea, applied externally for rheumatism. Rhizome is given to children to bite during teething, used in backbone ache, as tonic, emetic, and cathartic.

(8) Botanical name	*Peganum harmala* L.
Local name	Harmal
Family	Zygophyllaceae
Distribution in Pakistan	Skardu, D.I.Khan, Peshawar, Hassan Abdal, Quetta, Sibi, Zhob, Kalat, Nakran, and Lakki Marwat
Plant material of interest	Seeds
Organoleptic	Smell characteristic, taste is bitter
Macroscopic characters	Seeds are blackish brown, triangular, 2-3 mm long, and 1–1.5 mm in diameter.
Microscopical characters	Seed powder is brown, sclerenchymatous cells of testa parenchyma cells, and oil globules are present.
Pollen grains morphology	Pollens are suboblate-subprolate and tricolporate; sculpturing is striate-rugulate, reticulate, consisting of lumina and muri, perforate, pit diameter less than 1 *µ*m.
Traditional uses	Leaves are smoked to repel evil sight and mosquitoes and remove bad smells. It is a brain tonic, blood purifier, and remedy for tapeworm and is used in ear diseases.

(9) Botanical name	*Psoralea corylifolia* L.
Local name	Babchi
Family	Papilionaceae
Distribution in Pakistan	Peshawar, Rawalpindi
Plant material of interest	Seeds
Organoleptic	Odour aromatic similar to elemi, taste is bitter
Macroscopic characters	Seeds are greyish black, flattened, dotted oblong, dotted, reniform, and rough. 3–5 mm in length and 2-3 mm in diameter. Black seed coat/testa.
Microscopical characters	Seed powder is greyish black. Sclerenchymatous cells of testa, parenchymatous cells of calyx, and cotyledon are present. Epidermal cells and oil cells are also visible.
Pollen grain morphology	Scarbate and tectate with smooth surface.
Traditional uses	Used as blood purifier, antihelmintic, and expulsion of gases and piles.

(10) Botanical name	*Rauwolfia serpentina* L.
Local name	Choti Chandan, Chota Chand
Family	Apocynaceae
Distribution in Pakistan	Peshawar, Karachi
Plant material of interest	Root
Organoleptic	Odour is indistinct, earthy, reminiscent of stored white potatoes; taste is bitter.
Macroscopic characters	The root segments are 3–20 mm in diameter, subcylindrical to tapering. Externally light brown to greyish yellow, rough or wrinkled longitudinally, and smooth to the touch. Fracture is short but irregular.
Microscopical characters	Phloem parenchyma and calcium oxalate crystals are present, xylem parenchyma, wood fibres, and tracheids are present. Powder is brownish/reddish grey, and starch grains are present.
Pollen grain morphology	Foveolate with pits on the surface and scabrate.
Traditional uses	Used in high blood pressure, sleeplessness, and in scorpion bite.

(11) Botanical name	*Vetiveria zizanioides* L.
Local name	Khas
Family	Poaceae
Distribution in Pakistan	Rawalpindi
Plant material of interest	Whole plant
Organoleptic	Aromatic, taste characteristics
Macroscopic characters	Culms are present, leaf-blades, panicle contracted, raceme 5–7.5 cm long. Sessile spikelet, callus glabrous, glume spinulose, and awnless.
Microscopical characters	Trichomes and oil cells are present, and thin fibres, irregular cell structures, and yellow-brown cells are present.
Traditional uses	Used to cure fever, inflammation, and irritability of stomach and for aromatic properties.
